# Supraphysiological Levels of Quercetin Glycosides are Required to Alter Mineralization in Saos2 Cells

**DOI:** 10.3390/ijerph13050460

**Published:** 2016-04-29

**Authors:** Leslie A. Nash, Sandra J. Peters, Philip J. Sullivan, Wendy E. Ward

**Affiliations:** 1 Department of Health Sciences, Faculty of Applied Health Sciences, Brock University, 1812 Sir Isaac Brock Way, St. Catharines, ON L2S 3A1, Canada; Lnash051@uottawa.ca; 2 Centre for Bone and Muscle Health, Brock University, 1812 Sir Isaac Brock Way, St. Catharines, ON L2S 3A1, Canada; 3 Department of Kinesiology, Faculty of Applied Health Sciences, Brock University, 1812 Sir Isaac Brock Way, St. Catharines, ON L2S 3A1, Canada; speters@brocku.ca (S.J.P.); psullivan@brocku.ca (P.J.S.)

**Keywords:** flavonoids, hyperoside, mineral, rutin, osteoblasts, tea

## Abstract

Flavonoid intake is positively correlated to bone mineral density (BMD) in women. Flavonoids such as quercetin exhibit strong anti-oxidant and anti-inflammatory activity that may be beneficial for bone health. Quercetin, previously shown to positively influence osteoblasts, is metabolized into glycosides including rutin and hyperoside. We compared the effects of these glycosides on mineralization in human osteoblast (Saos2) cells. Administration of rutin (≥25 µM) and hyperoside (≥5 µM) resulted in higher mineral content, determined using the alizarin red assay. This was accompanied by higher alkaline phosphatase activity with no cell toxicity. The expression of osteopontin, sclerostin, TNFα and IL6, known stimuli for decreasing osteoblast activity, were reduced with the addition of rutin or hyperoside. In summary, rutin and hyperoside require supraphysiological levels, when administered individually, to positively influence osteoblast activity. This information may be useful in developing nutraceuticals to support bone health.

## 1. Introduction

Osteoporosis is defined by low BMD [1], a high risk of fragility fracture [2] and diminished quality of life [3]. Dietary strategies may help prevent osteoporosis by supporting peak bone mass as well as the maintenance of healthy bone after the growth period [4,5,6]. Flavonoids present in food and beverages such as tea [7], are emerging as potential bioactives for bone health [8]. Many studies show a positive association between flavonoid intake or habitual tea consumption and BMD in women [7,9,10,11,12,13,14]. One study showed an inverse association between flavonoid intake and a bone resorption marker [7] while most studies have not investigated whether bone formation, resorption or both are associated with habitual tea consumption and BMD. 

Flavonoids have a wide-range of biological effects due to anti-oxidant [15] and anti-bacterial properties [16] and may thereby benefit bone health [17,18]. Rutin (quercetin-3-*O*-rutinoside) and hyperoside (quercetin-3-*O*-galactoside) are flavonoids found in green [19], black [19] and rooibos tea [20] and other foods [21,22,23]. They are also derivatives from quercetin, another flavonoid found in a variety of food and beverages [24]. Quercetin increases alkaline phosphatase activity (ALP) in mouse osteoblasts [25], and BMD at the lumbar spine and femur in ovariectomized mice [26]. Derivatives of quercetin (quercetin-6-C-β-d-glucopyranoside) have been shown to increase ALP and mineral formation *in vitro*, and BMD in female rats [27]. Hyperoside was recently shown to promote differentiation in U2O2 and MG63 cells without promoting cellular death [28].

A few studies have shown that rutin and hyperoside stimulate osteoblast activity [28,29] but no study has directly compared the effects of these main metabolites of quercetin within the same model. The objective of this research was to identify and compare, in the same model using a wide range of concentrations, if the addition of rutin or hyperoside, results in greater mineral content in Saos2 cells. Structure-function relationships between these two flavonoids were also assessed. 

## 2. Materials and Methods 

Saos2 cells [30] were purchased from Cedarlane (Supplier ATCC, Burlington, ON, Canada). HAM-F12, 10× Trypsin, PBS, fetal bovine serum (FBS), antibiotic-antimycotic and flasks were purchased from Lonza (Mississauga, ON, Canada). Rutin trihydrate (≥97%) and 3-(4,5-dimethy-2-lthiazolyl)-2,5-diphenyl-2H-tetrazolium bromide were purchased from Alfa Aesar (Reston, VA, USA) and hyperoside (≥98%) from Aktin Chemicals (Sichuan, China). Human Milliplex bone magnetic bead panels for bone metabolism were purchased from EMD Millipore (Billerica, MA, USA) and read by xPONENT software on Magpix Luminex (Austin, TX, USA). Optical density (OD) measurements were analyzed using a BIO-TEK Synergy HT Multi-Detection Microplate Reader (Winooski, VT, USA). Other chemicals were purchased from Sigma-Aldrich (Oakville, ON, USA).

Cells were maintained and differentiated as previously described [31]. Following one week of differentiation in plates, differentiation cell media was supplemented with 0.01 M β-glycerophosphate (day 8) until day 21 (Media 3) in addition to either vehicle (0.1% DMSO) or flavonoid dissolved in DMSO to 0.1%.

For all experiments, a DMSO control was compared to flavonoid treated cells. All experiments were normalized to cell count using Trypan Blue staining. 

### 2.1. Mineral Content

Mineral content was quantitatively assessed using the alizarin red assay [31]. Cells plated at 5 × 10^3^ cells/well in 24 well plates. On day 21 cells were fixed in 4% paraformaldehyde and kept at 4 °C for 24 h. Following, cells were washed with PBS and replaced with 0.04 M alizarin red S stain for 20 min on a plate shaker at room temperature. Cells were washed with PBS. Extraction required the addition of a 10% cetylpyridinium chloride solution for 1 h at room temperature. Mineral was quantified by measuring OD at 550 nm. 

### 2.2. ALP Activity

Cells were grown in 12 well plates at 1 × 10^4^ cells/well and lysed by mechanically homogenizing cells using ice cold 0.05% Triton X-100 solution dissolved in 0.05 M Tris-HCl. Cells were centrifuged at 10,000 rpm at 4 °C for 10 min. Cell lysates were introduced to p-nitrophenyl phosphate. P-nitrophenol production was monitored over 5 min and was stopped by the addition of 0.5 M NaOH. The OD was measured at 410 nm and normalized to protein concentration using the Bradford assay [32,33]. Cells were tested on days 3 (day 11), 7 (day 15) and 10 (day 18) after the addition of Media 3 and flavonoid. 

### 2.3. Cell Activity

Mitochondrial reductase activity was measured by the reduction of 3-(4,5-dimethyl-2-thiazolyl)-2,5-diphenyl-2H-tetrazolium bromide (MTT). Cells were plated in 96 well plates at 1 × 10^4^ cells/well, and allowed to adhere over 24 h. Media was replaced every 24 h. MTT reduction was measured at 24, 48, and 72 h following the addition of the flavonoid. Cells were washed three times with PBS and then supplemented with media sans flavonoid, with 5 mg/mL MTT for 4 h. Media was removed and crystals were dissolved in 0.04 M HCl-isopropanol. OD was taken at 570 nm with a reference measurement at 610 nm. 

### 2.4. Cell Toxicity

Lactate dehydrogenase (LDH) was measured in cell media to quantify cell death. Cells were plated following the MTT protocol. LDH was measured by an *in vitro* toxicology assay kit from Sigma-Aldrich (Catalogue ID: Tox7). Media with flavonoid was replaced every 24 h and cells were harvested at 12, 24 and 48 h to be analyzed by Tox7 kits. 

### 2.5. Osteoblast Regulatory Proteins

Levels were chosen based on their ability to promote significant mineralization without inducing toxicity. Cells were plated at 500,000 cells in 75 cm^2^ flasks and allowed to adhere for 24 h. The addition of media 2 and media 3 followed differentiation guidelines indicated above. When the media was changed to media 3 (day 8), it was accompanied with the addition of vehicle (DMSO) or flavonoid. The cells, once exposed to media 3, were left for 3 days prior to media extraction (day 11). Media was examined early into mineralization to identify immediate changes in protein markers for osteoblast activity. Extraction of media at this time point, correlates with results obtained for ALP (day 3 into mineralization or day 11 total). All protein levels, OPG, OPN, SOST, IL6, TNFα were determined from a standard curve created with purified protein dissolved in media 3, using the ELISA reader. Cell media was analyzed using a commercial ELISA based assay for TNFα, IL6, SOST, OPG and OPN (Millipore, HBNMAG-51K, Billerica, MA, USA). Media was chosen to analyze as opposed to cell lysates to mimic the clinical scenario in which bone markers released into circulation are measured. Treated media was compared to cells exposed to vehicle (0.1% DMSO). In addition, media was added to each well within the ELISA to account for any background noise. 

### 2.6. Statistics 

Measurements of mineral content consisted of *n* = 6, with each sample corresponding to a separate experiment of three wells averaged together. Measurements for ALP, MTT and LDH consisted of *n* = 6 per time point, with each sample measured in triplicate. Protein measurements were completed using *n* = 4, with each sample measured in triplicate. Measurements are reported as an average ± SEM. 

Statistical analyses were performed using IBM SPSS Statistics 21, utilizing 1 way-ANOVA with vehicle (rutin, hyperoside, or DMSO control) as the independent variable (IV). The dependent variable (DV) was expressed as per cent DMSO control and was a function of mineral, ALP activity, MTT reduced, LDH released or concentration of protein. If the 1 way-ANOVA was statistically significant (*p* < 0.05), *post-HOC* analysis was used to identify differences across groups (*p* < 0.05). Data was checked for homogeneity of variance. When homogeneity of variance was not statistically different (*p* > 0.05), *post-HOC* analysis utilized Bonferroni *t*-test. Where homogeneity of variance was statistically different across groups (*p* < 0.05), Games Howell *t*-test was utilized.

## 3. Results

### 3.1. Mineral Content

**Figure 1:**
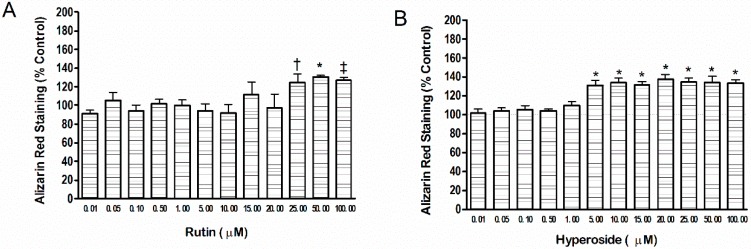
Mineral content produced by addition of (**A**) rutin or (**B**) hyperoside. Mineral content was measured by alizarin red staining. Significant differences are indicated by: † *p* < 0.05, ‡ *p* < 0.01, * *p* < 0.001 compared to control. Each bar represents *n* = 6.

Rutin (25 µM, 50 µM and 100 µM) resulted in higher mineral content (*p* < 0.05, *p* < 0.001, *p* < 0.01, respectively) compared to control (Figure 1). Hyperoside resulted in significantly higher mineral content at lower doses (5 µM through 100 µM, *p* < 0.001) compared to control. 

### 3.2. ALP Activity

**Figure 2:**
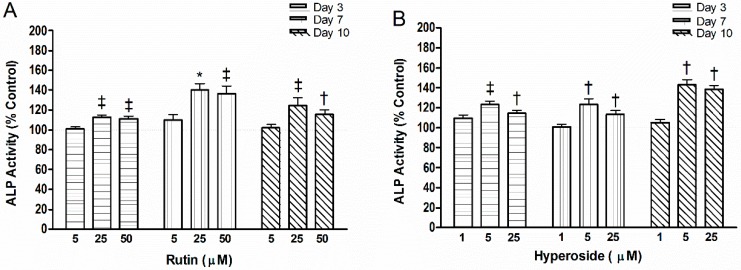
Alkaline phosphatase activity (ALP) activity with addition of (**A**) rutin or (**B**) hyperoside. Activity was measured by the rate of hydrolysis of p-nitrophenyl phosphate. Significant differences are indicated by: † *p* < 0.05, ‡ *p* < 0.01, * *p* < 0.001 compared to control within a day. Each bar represents *n* = 6.

Two doses in which rutin or hyperoside resulted in higher mineral content were chosen along with one dose that did not (served as a negative control, Figure 2). Rutin (25 µM, 50 µM) resulted in higher ALP activity than control at day 3 (*p* < 0.01, *p* < 0.01, respectively), day 7 (*p* < 0.01, *p* < 0.01, respectively) and day 10 (*p* < 0.01, *p* < 0.05, respectively). Exposure to hyperoside (5 µM, 25 µM) resulted in higher ALP activity than control at day 3 (*p* < 0.01, *p* < 0.05, respectively), day 7 (*p* < 0.05, *p* < 0.05, respectively) and day 10 (*p* < 0.05, *p* < 0.05, respectively). 

### 3.3. Cell Mitochondrial Activity

**Figure 3:**
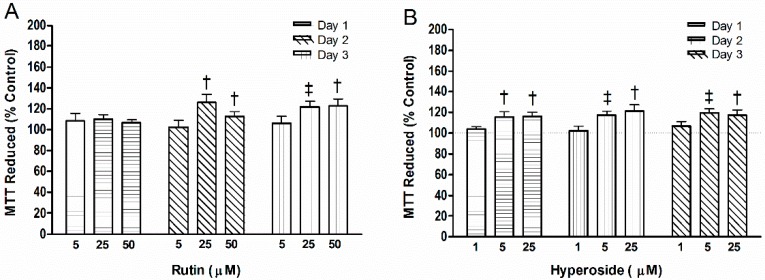
3-(4,5-dimethyl-2-thiazolyl)-2,5-diphenyl-2H-tetrazolium bromide (MTT) levels reduced with addition of (**A**) rutin or (**B**) hyperoside. Activity was measured by the reduction of MTT. Significant differences are indicated by: † *p* < 0.05 and ‡ *p* < 0.01 compared to control within a day. Each bar represents *n* = 6.

Rutin (25 µM, 50 µM) increased mitochondrial activity after 48 h (*p* < 0.05, *p* < 0.05, respectively) and 72 h (*p* < 0.01, *p* < 0.05, respectively) compared to control (Figure 3). At 24 h, hyperoside (5.0 µM, 25.0 µM) increased mitochondrial activity (*p* < 0.05, *p* < 0.05, respectively) compared to control and was maintained for 48 h (*p* < 0.01, *p* < 0.05, respectively) and 72 h (*p* < 0.01, *p* < 0.05, respectively).

### 3.4. Cell Toxicity

**Figure 4:**
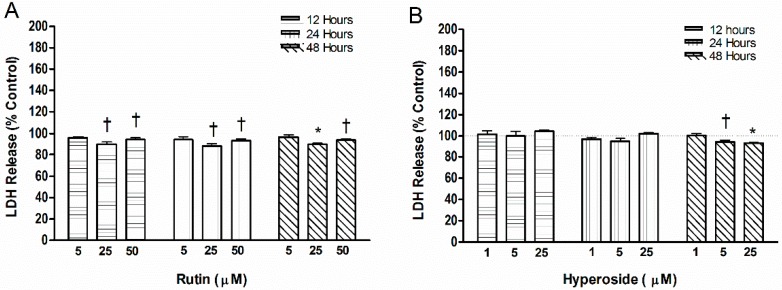
Lactate dehydrogenase (LDH) release by addition of (**A**) rutin or (**B**) hyperoside. LDH activity was measured by the reduction of NAD^+^ to aid in the conversion of a tetrazolium dye to a formazan crystal. Significant differences are indicated by: † *p* < 0.05, and * *p* < 0.001 compared to control within a day. Each bar represents *n* = 6.

After 12 h of the initial flavonoid addition, rutin (25.0 µM or 50.0 µM) resulted in lower levels of LDH (*p* < 0.05, *p* < 0.05, respectively) compared to control (Figure 4), and was maintained at 24 h (*p* < 0.05, *p* < 0.05, respectively) and 48 h (*p* < 0.001, *p* < 0.05, respectively). Hyperoside did not alter LDH levels after 12 and 24 h of supplementation. After 48 h, hyperoside (5.0 µM, 25.0 µM) demonstrated lower LDH levels (*p* < 0.05, *p* < 0.001, respectively) than control. 

### 3.5. Quantification of Regulatory Proteins in Osteoblasts

Rutin and hyperoside resulted in lower levels of TNFα (*p* < 0.001, *p* < 0.001), IL6 (*p* < 0.001, *p* < 0.001), OPN (*p* < 0.001, *p* < 0.001), OPG (*p* < 0.01, *p* < 0.01), and SOST (*p* < 0.001, *p* < 0.001) than control. Direct comparison of rutin (50 µM) and hyperoside (25 µM) showed that rutin resulted in a greater reduction in TNFα (*p* = 0.035), IL6 (*p* < 0.002) and OPG (*p* < 0.001) compared to hyperoside. Normalizing the protein data to the amount of flavonoid added (per mol) using Equation (1) showed that hyperoside induced greater changes than rutin on a per mol basis (see Table 1).

[(ProteinFlavonoid – ProteinControl)/(molesFlavonoid)]
(1)

## 4. Discussion

This study, the first to directly compare effects of two different glycosides of quercetin in a bone cell model, has shown that rutin (25–100 µM) and hyperoside (5–100 µM) stimulate a higher mineral content that is accompanied by higher alkaline phosphatase activity and cell mitochondrial activity with no cell toxicity. Moreover, expression of OPN, SOST, TNFα and IL6, known stimuli for decreasing osteoblast activity, were reduced with the addition of rutin or hyperoside. Of note is that basal levels of TNFα and IL6 were likely elevated because the Saos2 cell line is cancerous whereas unstimulated osteoblasts may have lower levels of these cytokines that may remain unchanged with the intervention. While rutin and hyperoside have not been previously studied in human Saos2 cells, in agreement with our findings, rutin has been shown to enhance mitochondrial activity in mouse bone marrow mesenchymal stem cells [29]. Similarly, hyperoside has been shown to increase ALP activity in a rat osteosarcoma cell line but mineral was not measured [34]. 

The down-regulation of TNFα and IL6 by rutin and hyperoside is important as high oxidative stress can inhibit differentiation and activity of osteoblasts and increase osteoclast activity [35,36] and such effects can be inhibited with pretreatment with antioxidants [37]. We also showed that OPG expression was altered by rutin and hyperoside. While OPG does not directly change osteoblast behavior and thus mineral formation, it is involved in bone turnover in the presence of osteoclasts. Specifically, it is the ratio of RANKL to OPG that determines osteoclastogenic potential, and since RANKL was not measured in this study, the effect of this ratio should be evaluated in a future study. Wnt3 inhibitors (DKK-1 and -2) reduce OPG expression in osteoblasts [38] and the production of the Wnt inhibitor SOST would lead to an increase in OPG levels. However, the addition of rutin and hyperoside resulted in lower levels of OPG. This may be an artifact of the Saos2 cell line as it is a cancerous cell line and high OPG levels are a marker for cancer. OPG acts as a decoy ligand for tumor necrosis factor related apoptosis ligand (TRAIL) [39] that is primarily in cancerous cells and stimulates apoptosis [39]. Also, it was previously shown that rutin (≥50 µM) increases OPG in mouse osteoblasts [29] Therefore, the difference seen between these two studies may be due to differences in OPG machinery found within the particular cell line. High OPG levels in a human osteosarcoma cell line are positively correlated with TNFα [40] and suggest that inflammatory cytokines may aid in tumor proliferation by stimulating the production of OPG. As the addition of rutin and hyperoside reduced both TNFα and IL6, and previous work has shown that levels of TNFα are related to levels of OPG in cancer [40], OPG may have decreased as a result of lower levels of TNFα and IL6.

OPN and SOST are markers of mineral inhibition. OPN is predominately recognized as an inhibitor for mineralization [41,42,43]. OPN knock-out mice have favorable bone outcomes: reduced osteoclast motility and bone resorption as well as higher trabecular bone area, and trabecular thickness with less trabecular spacing [44]. SOST inhibits the Wnt pathway and regulates bone formation [45]. Our study is the first to show that rutin and hyperoside down-regulate Wnt inhibitors in osteoblasts. Previous work in mouse osteoblasts has shown that the addition of rutin (≥25 µM) increased transcriptional levels of OPN [29] however protein levels were not measured. Another study demonstrated that carnosol from rosemary reduced OPN and IL6 in osteoblasts [46]. While our data demonstrates changes to osteoblast and inflammatory markers early into the mineralization phase, future work should examine changes in protein expression of these markers over differentiation, early mineralization and during the final end point. Establishment of these parameters at various time points would help determine whether greater or lower protein expression is transient, or whether changes occur with alterations in dose and/or time. The Saos2 cell line has been documented as an osteoblast line that mimics primary cells very closely [47,48]. However, comparison of data from this and other commonly used cancerous osteoblast cell lines (U2OS, UMR106, UMR, ROS, MG63) to primary cell lines is also necessary to determine key differences in bone metabolic markers after addition of bioactives.

A study using ovariectomized rats showed preservation of BMD in both total and distal femur with a diet of 0.25% rutin [49]. Higher femur peak load and a reduction in deoxypyridinoline, a marker of bone resorption was also observed [49]. However, ingestion of rutin leads to a variety of metabolites that may produce these biological effects, in which the authors identified quercetin and isorhamnetin in the low micromolar range [49]. Rutin has also been detected in human plasma after consuming tea [50] and pure rutin tablets [51] from low to high nM range. In contrast, hyperoside was not detectable in plasma after consumption of tea [50] but was detected in rat plasma in a dose-dependent manner after consumption of hyperoside tablets, but was quickly cleared by the hepatic system [52]. Hyperoside may therefore be a good candidate for encapsulation or chemical enhancement to improve its bioavailability. The bioavailability of rutin can be improved by conjugating it to 2-hydroxy-propyl-β-cyclodextrinin [53]. Bioavailability can also be improved by the presence of other bioactives that alters solubility and stability prior to absorption. Bioavailability of hypericin increases by 34% in the presence of hyperoside [54]. The mid to high micromolar ranges of these compounds, needed for biological effects, are not achieved through consumption of food and beverage. Thus, these results suggest studying the combination of flavonoids as opposed to individual flavonoids, as interactions of flavonoids with other flavonoids or food components may contribute to greater bioavailability.

As the addition of rutin or hyperoside led to an overall increase in mineral production, a dose-dependent effect was not observed. This may suggest that the individual flavonoids do not directly impact mineralization through binding to receptors, but instead act indirectly by providing osteoblasts with a more stable, enriched environment for mineralization to occur, by limiting pro-inflammatory cytokines that are known to be harmful to the mineralization process. Therefore, instead of a dose-response, a threshold of antioxidant activity must be reached to identify any benefit. This finding requires investigation in future studies.

Effective flavonoid concentrations may vary due to structure-function relationships. Rutin has greater hydrophobicity that modulates interactions at water-lipid interfaces and its ability to act as an antioxidant [55]. Quercetin [56,57] and rutin [57] have been shown to have high antioxidant potential. While we did not directly measure the antioxidant activity of rutin in our system, we indirectly showed this through down regulation of pro-inflammatory markers, TNFα and IL6. The C3 hydroxyl on the flavonoid backbone largely contributes to the antioxidant activity because of its close proximity to the 4C keto and the conjugated 2-3C leading to the aromatic B ring—this helps stabilize free radicals [58]. The loss of this C4 hydroxyl, due to the addition of sugars, results in significantly lower antioxidant capacity [56]. The addition of two sugars (*i.e.*, rutin) creates steric hindrance, further impacting the antioxidant capacity. The antioxidant capacity of quercetin is stronger than its glycosides [56,57]. The substitution of sugars at the C3 position (*i.e.*, rutin) in comparison to their aglycone (quercetin) with a hydroxyl group at that position, inhibited superoxide radical scavenging activity of flavonoids [59]. However, this free hydroxyl located on C3 can allow for quercetin to undergo auto-oxidation. Thus, quercetin can have pro-oxidant activity by increasing the levels of superoxide radicals and hydrogen peroxide within a local environment. Yet because of the disaccharide on C3 of rutin, it maintains potent antioxidant activity without acting as a pro-oxidant. This disaccharide also increases the hydrophobicity of rutin, allowing for easier uptake into membranes [59]. 

Isoquercetin is another glycoside of quercetin and has been shown to be toxic at levels as low as 10 µM in Saos2 cells [60]. Hyperoside and isoquercetin are epimers; the hydroxyl on the 4th carbon of the sugar ring is pointed in a different direction, thereby making them diastereomers. This difference results in a change in activity, and increases toxicity. The glucose ring of hyperoside may shield the C4 keto group on the flavonoid backbone, allowing a hydrogen bonding opportunity between the C4 hydroxyl on the galactoside and the keto group on the backbone. In contrast, the glucose of isoquercetin has its ring pointed away, leaving the C4 keto group open. This keto group is likely a key structure for pro-oxidant activity as it has the potential to create quinone, capable of generating reactive oxygen species [61]. This production of reactive oxygen species was identified with quercetin but failed to occur with rutin [62]. Flavonoids that are too strong of an antioxidant can have pro-oxidant activities. The position of a glycoside on the flavonoid can result in cellular death. 

## 5. Conclusions 

In closing, the levels of rutin and hyperoside required to enhance mineralization are greater than those present naturally in food, and the differing effects of these two metabolites highlight the need to consider whether it is provided in isolation or within a food source. Moreover, identification of key structural changes that lead to positive effects in osteoblast physiology may aid in the development of nutraceuticals that target bone. Future study in primary osteoblasts is warranted because Saos2 cells are derived from a primary osteosarcoma and may have genetic and epigenetic modifications that could alter their biology.

## Figures and Tables

**Table 1:** Concentration of regulatory proteins involved in osteoblast activity.

Condition	TNFα	IL6	OPN	OPG	SOST
Control (pg/mL)	0.43 ± 0.01	3.24 ± 0.08	247.50 ± 10.71	1659.25 ± 107.78	810.00 ± 22.15
50 µM Rutin (pg/mL)	0.20 ± 0.02 ^a,^*	1.36 ± 0.09 ^a,^*	126.21 ± 10.78 *	431.25 ± 11.37 ^a,^^‡^	572.00 ± 19.07 *
Rutin (pg/mL/mol) **^#^**	−46	−376	−24,258	−245,600	−47,600
25 µM Hyperoside( pg/mL)	0.25 ± 0.01 ^b,^*	1.90 ± 0.06 ^b,^*	145.75 ± 2.75 *	709.25 ± 25.28 ^b,^^‡^	542.75 ± 10.01 *
Hyperoside pg/mL/mol **^#^**	−72	−536	−40,700	−380,000	−106,900

Data are expressed as mean ± SEM, *n* = 4; Significant differences in values, from control, are indicated by: **^‡^** *p* < 0.01 and * *p* < 0.001 within a column. Significant differences (*p* < 0.05) between rutin and hyperoside are indicated by ^a^ *vs.* ^b^ within a column. **^#^** The ability for a mol of flavonoid to change the expression of protein relative to DMSO control.

## Abbreviations

The following abbreviations are used in this manuscript:

ALP	alkaline phosphatase activity
BMD	bone mineral density
DMSO	dimethylsulfoxide
IL6	Interleukin 6
LDH	lactate dehydrogenase
MTT	2-(4,5-Dimethylthiazol-2-yl)-2,5-diphenyltetrazolium bromide
OD	Optical density
OPG	osteoprotegerin
OPN	osteopontin
SOST	sclerostin
TNFα	tumor necrosis factor alpha
